# Lower limb joint repair and replacement: an overview

**DOI:** 10.12688/f1000research.17200.1

**Published:** 2019-12-19

**Authors:** Rocco Aicale, Nicola Maffulli

**Affiliations:** 1 Department of Musculoskeletal Disorders, School of Medicine and Surgery, University of Salerno, Baronissi, Italy; 2 Clinica Ortopedica, Ospedale San Diovanni di Dio e Ruggi D'Aragona, Salerno, Italy; 3 Barts and the London School of Medicine and Dentistry, Centre for Sports and Exercise Medicine, Mile End Hospital, Queen Mary University of London, London, UK; 4 School of Pharmacy and Bioengineering, Faculty of Medicine, Institute of Science and Technology in Medicine, Guy Hilton Research Centre, Keele University, Stoke-on-Trent, UK

**Keywords:** Joint Arthroplasty, Total hip Arthroplasty, Total Knee Arthroplasty, Total Ankle Arthroplasty

## Abstract

In the last few years, much has been published concerning total joint arthroplasty, and debates and discussions to new questions and points of view started many years ago. In this commentary, we report the latest evidence of best practice in the field of lower limb arthroplasty; this evidence is based on a literature search conducted by using PubMed and Scopus databases with a time limit of five years. We found novel evidence regarding cemented and not cemented implant, implant design, anticoagulant use, tourniquets, and other aspects of joints replacement surgery that we consider a common part of modern orthopedic practice. We specifically focus on lower limb joint replacement.

## Introduction

During the past few years, several advances in joint arthroplasty have been reported. For the purpose of this article, we evaluated articles published in peer-reviewed journals during the last five years. We have considered many studies regarding best practices that may improve outcome and prevent complications and new studies regarding designs and materials of the devices used in this type of surgery. Many of these topics remain controversial in routine orthopedic practice. We report the latest available scientific evidence regarding lower limb arthroplasty.

## Total hip replacement: new evidence

During the last few years, there has been a great deal of interest in conventional cementless and ultrashort stems. Conventional cementless femoral stems demonstrated a good rate of clinical and radiographic performance at long-term follow-up ^1– 3^. Kim *et al*., in a level I study, compared the use of ultrashort and conventional cementless femoral stems in patients younger than 55, with a mean follow-up of 11.8 years, resulting in absence of significant differences of outcome scores ^1^. Another randomized controlled trial, in which the follow-up was 24 months, reported similar results ^4^.

To provide intraoperative options of femoral neck length and offset and maximize mechanics and stability during hip arthroplasty, the concept of neck modularity was introduced ^5^. Langton *et al*. ^6^ investigated head-neck taper failure in a contemporary metal-on-metal arthroplasty system. Great variation in the manufactured surface finish of the female taper surface was identified, and the female taper surface roughness was associated with taper wear ( *P* <0.001) ^6^.

Variations in manufacturing tolerance play a major role in the development of fretting, corrosion, implant failure, and the production of serum metal ions. Serum metal ion levels have been investigated, detecting taper corrosion-related pseudotumors in patients with dual taper modular total hip arthroplasty (THA) components ^7, 8^. In particular, cobalt levels of 2.8 µg/L have an 88% sensitivity and 32% specificity in predicting pseudotumors on magnetic resonance imaging. The same study ^7^ stressed that the absence of symptoms does not exclude the presence of adverse local tissue reactions (ALTRs) and an elevated cobalt level; an elevated cobalt-to-chromium ratio of 3.8 was associated with the presence of pseudotumors in asymptomatic patients and symptomatic patients ^7, 9^.

All combinations of bearing surface have advantages and disadvantages, and recently the use of some of them (for example, metal-on-metal and ceramic-on-metal) has markedly decreased. ****Recent technological developments in the field of polyethylene and ceramics have impacted on the risk of fracture and the rate of wear ^10, 11^. Probably, the optimal bearing surface for a given patient needs to be decided by analyzing specific implications for each patient. For example, the long-term performance of cross-linked polyethylene (XLPE) may not be relevant for patients with a life expectancy of less than 15 years but should be taken into account if a patient is, for example, 35 years old. In such a patient, the trade-off between long-term low wear from a ceramic-on-ceramic bearing may outweigh the small risk of fracture and squeaking ^12^.

The stems are produced by various manufacturers, span various taper geometries, and are made using different materials, such as cobalt-chromium (Co-Cr) alloy and titanium (Ti) alloy or metal-on-polyethylene. ALTRs secondary to head-neck taper corrosion in THA have been reported ^13^. The modular neck femoral stems facilitate the intra-operative restoration of patient anatomy, but corrosion at the neck-stem junction has also been observed: patients with Co-Cr modular necks typically present with ALTRs due to taper corrosion ^14^, whereas those with Ti necks more commonly present with neck fracture ^14, 15^. These findings may result from the greater strength and fatigue resistance of Co-Cr alloy in comparison with those of Ti alloy.

Recently, many studies investigated the complication of THA following an increase in the use of joint replacement worldwide ^5^ and focused on the risk of revisions, infections, and prevention of infection and deep vein thrombosis (DVT) ^16, 17^.

Bozic *et al*. reported good results in the midterm (5 to 7 years) regarding revision risk in arthroplasty patients from the period of 1998 to 2002 to that of 2008 to 2010, during which there was a 14% overall reduction of revision ^18^.

A severe complication is periprosthetic joint infection (PJI), which results in increased costs, lengthy hospitalizations, and substantial patient morbidity. An economic analysis of tertiary care centers showed a threefold increase in costs and a higher number of readmissions for THA-treated patients who experienced a PJI compared with those with no infection ^19^. In this context, more aggressive pre-operative prevention and management of diabetes may help to prevent this complication.

Two recent studies showed that intra-articular hip injections within 3 months before THA was a risk factor for PJI in the first operative year, increasing the infection incidence from approximately 0.5 to 1.0% ^20, 21^. In particular, corticosteroid injections should be discouraged within 3 months of a planned THA procedure. Some studies reported incidences of infection of 2.41% at 3 months (odds ratio, 1.9; *P* = 0.004), significantly higher compared with the control group, and 3.74% at 6 months (odds ratio, 1.5; *P* <0.019); the incidence of infection was not significantly higher compared with patients who did not undergo hip injection within 3 months before THA ^20, 21^.

Two reports demonstrate that screening and decolonization improved surgical site infection (SSI) rates, but decolonization following nasal swabs without screening was effective and resulted in cost savings ^22, 23^. Furthermore, cost savings can be realized with the substitution of nasal povidone-iodine swabs with mupirocin topical nasal antibiotic ^23^. Treating all patients without testing also eliminates the logistical difficulties of screening.

Because 30% of infections in THA resulted from Gram-negative organisms, recent studies examined, in addition to a first-generation cephalosporin, the use of vancomycin and other antibiotics ^24, 25^. Bosco *et al*. used Gram-negative antibiotic prophylaxis for patients with THA ^26^, and the infection rate decreased from 1.2 to 0.6% using pre-operative gentamycin with cefazolin. However, additional risks of this practice are antibiotic toxicity, increased costs, and the development of resistance ^25^.

Discharge to home remains an interesting topic following joint replacement surgery. A study about post-discharge adverse event risk in patients undergoing elective THA concluded that discharge home reduces the risk of adverse events compared with discharge to a skilled nursing facility or inpatient rehabilitation facility ^16^. Hence, home discharge, when feasible, is the more preferable destination compared with an inpatient setting.

An Enhanced Recovery After Surgery (ERAS) program has been proposed to reduce post-operative morbidity and length of hospital stay for patients who underwent hip and knee arthroplasty. Several studies on ERAS show good results with no increase of short-term complications and readmission rates ^27^, and similar results have been reported in other studies ^28, 29^.

Symptomatic DVT and pulmonary embolism (PE) are important complications of major orthopedic surgery, and in the last decade, the use of aspirin for their prophylaxis has been reported in multiple studies. Recent studies support the prophylaxis with aspirin making risk stratification of patients, demonstrating the equivalence of aspirin use compared to aggressive anticoagulant therapy, with a lower risk of major bleeding events ^30– 32^. However, it is not universally agreed which patients constitute major risk ^33^.

To reduce the use of allogeneic blood transfusions and blood loss in the post-operative period, research in blood management following total joint arthroplasty has focused on various modalities, including the use of tranexamic acid (TXA) ^34^. TXA is a synthetic amino acid derivative of lysine that inhibits binding of fibrin to plasminogen, preventing degradation of the fibrin clot ^35^. A recent meta-analysis reports strong evidence on the use of TXA to reduce blood loss and the risk of transfusions following THA ^36^, but no evidence of difference outcomes following different administration modalities, single or multiple doses, are reported supporting only the administration of a pre-incision low dose of TXA.

Great interest surrounds the use of different surgical approaches for THA. In particular, the direct anterior approach has become popular and its proponents claim superior results for improved kinematics and better long-term outcomes following its use for THA ^37^. This approach is commonly used in pediatric surgery for developmental dysplasia of the hip and femoroacetabular impingement. However, the available scientific literature does not support the above claims, and it remains controversial. Recent studies reported a modest improvement in early recovery with similar rates of complications after 6 weeks ^38^. In contrast, many reports describe an increased risk of peri-operative complications associated with the direct anterior approach. Furthermore, there are many disadvantages of the anterior approach, including a steep learning curve and the need for further release of tendon and capsule ^39, 40^ and the difficulty of using it in obese patients ^41^. Indeed, compared with other approaches, the anterior approach has been associated with a higher rate of wound complications. Sibia *et al*. evaluated 700 patients: 75 (11.5%) experienced wound complications requiring additional intervention, of which 13 (2%) required a reoperation ^42^. The main risk factors seem to be obesity and diabetes. In particular, a body mass index (BMI) of less than 28 would minimize these risks ^43^.

Using data from the Nationwide Inpatient Sample, Menendez *et al*. found that in-hospital rate of dislocation after elective THA increased from 0.025% to 0.15% from 2002 to 2011 ^44^. Another study reported a 0.92% dislocation rate (eight out of 871 hips) after direct anterior THA; the first dislocation occurred in the early post-operative period (a mean of 3 weeks) for six of the hips ^45^. After the first month, this risk essentially disappeared.

Another report on the risk of dislocation, in which more than 2.100 THA patients with the direct anterior approach were evaluated, showed no difference in the dislocation rate between these patients (0.84%) and a who underwent THA with posterior approach (0.79%) in term of propensity score ^46^. ****

## Total knee replacement: new evidence

The gold-standard treatment for patients with end-stage knee arthritis is total knee arthroplasty (TKA). In patients eligible for unilateral TKA following failure of non-operative treatment, Skou *et al*. ^47^ showed that TKA resulted in greater pain relief and functional improvements compared with non-operative treatment alone. However, controversy regarding optimal technique, instrumentation, and prosthesis design remains.

Several factors may increase the risk of infection in total joint replacement of the lower limb. In particular, for TKA, a recent systematic review of observational studies found that patients with diabetes have an increased incidence of complications, including deep infection, DVT, and aseptic loosening ^48^. A meta-analysis of observational studies evidenced a higher rate of deep infection and revision in obese patients compared with non-obese patients ^49^. Furthermore, the presence of peripheral vascular disease is a risk factor for deep infection ^50^ and wound-healing problems ^51^. The evidence for the management of peripheral vascular disease pre-operatively is limited, and no data support specific interventions to optimize TKA outcomes. However, as reported by the American Academy of Orthopaedic Surgeons guidelines, ankle brachial pressure of less than 0.9 should trigger a referral for vascular assessment and possible intervention before TKA. In these patients, the use of intra-operative tourniquet is generally not recommended. The presence of these factors should trigger referral to a specialty team to optimize the patient for surgery ^52, 53^.

No significant differences in terms of clinical outcomes, pain, or complications rate have been found among the various approaches to the knee ^54^. Furthermore, no significant evidence supports the use of high-flexion TKA ^55^, single-radius ^56^, mobile-bearing ^57^, or cementless ^58^ knee designs. At present, cement remains the gold standard and provides reliable fixation in various prosthetic total knee replacement designs ^59^. Preliminary evidence is emerging regarding a cemented stemmed tibial component that may slightly improve reliability and clinical outcomes (that is, the Knee Society Score and the Knee injury Osteoarthritis Outcome Score [KOOS]) in obese patients ^60^.

The management of the patella during TKA remains controversial. The role of patellar eversion is currently debated in terms of time to leg lift and active range of motion. A 2016 study reported a negative effect on early knee function ^61^, whereas another study reports the opposite result ^62^. Resurfacing of the patella generates much debate. Aunan *et al*. ^63^ reported no differences in KOOS and visual analogue scale (VAS) scores between patellar resurfacing compared with not resurfacing. The common practice of patellar denervation by electrocautery does not appear to impact on the rate of post-operative pain or knee function ^64^.

The reproduction of normal anatomy has always led to the exploration of alternative alignment paradigms in TKA. Two recent studies found no differences in terms of function and survivorship using kinematic alignment compared with mechanical axis alignment ^65, 66^.

Survivorship of TKA during the past 20 years has greatly improved, but polyethylene wear and eventual aseptic loosening remain major causes of revision. Developments in the production and processing of polyethylene inserts have decreased wear rates ^67^. However, development of sequentially irradiated or annealed polyethylene and vitamin E polyethylene were shown to retain mechanical properties of highly cross-linked polyethylene with lower wear rates and fewer free radicals ^68^, but problems with strength and fatigue resistance remain. Oxidized zirconium knee arthroplasties have been proposed to provide durability of metal component, reducing the coefficient of friction of the ceramic surface. This type of material has been associated with decreased wear rates ^68, 69^.

The use of tourniquet results in benefits in terms of total blood and hidden blood loss without significant effects on post-operative transfusions ^70^. However, it can be associated with muscle ischemia and atrophy ^71^ and delayed functional recovery ^72^. Using a cemented implant during TKA without a tourniquet did not affect the quality of fixation compared with controls ^73^.

In TKA, as in THA procedures, the use of TXA has been investigated. A recent meta-analysis concluded that its use is safe and reduces blood loss during surgery and the need for blood transfusions after primary TKA, however, no TXA formulation, dosage, or number of doses provide to clearly improved blood-sparing properties ^74^. Moderate evidence supports pre-incision administration to improve TXA efficacy ^74^.

Venous thromboembolic prophylaxis (VTP) is essential for all patients undergoing TKA. The incidence of PE within 30 days was reported to be 0.50%. Risk factors associated with PE were age of more than 70 years, higher BMI, female sex, and undergoing an arthroplasty ^75^.

A recent systematic review evaluated the effectiveness of various agents used for VTP: the choice of anticoagulant did not significantly affect the rates of symptomatic venous thromboembolism, DVT, or PE over placebo, increasing the risk of bleeding ^76^. Recently, the use of aspirin for VTP in low-risk individuals has gained popularity given its convenience and low cost ^77^.

## Total ankle replacement: new evidence

Arthrodesis of the ankle is the most frequent salvage operation performed for advanced arthritis, although the volume of total ankle arthroplasty (TAA) has increased over the past decade ^78– 80^.

The most important theoretical benefit of TAA compared with arthrodesis is the improvement in gait from preservation of ankle motion. Two recent studies compared TAA with ankle arthrodesis: both procedures resulted in improved gait post-operatively but failed to normalize the gait pattern ^80, 81^.

At intermediate follow-up, clinical outcomes of TAA and ankle arthrodesis were comparable in a retrospective study, and reoperation rate and major complications were higher in the ankle replacement cohort ^82^.

Pain and function improvements following TAA were reported to be similar between fixed and mobile-bearing devices, and peak plantar flexion moment and 36-item Short Form Health Survey (SF-36) scores were better in the first group, whereas the second group had greater improvement in VAS scores ^38^.

TAA seems to be effective to improve pain and function in all types of ankle arthritis. Ramaskandhan *et al*. reported similar clinical improvements at two-year follow-up in patients treated with three-component Mobility Total Ankle System (DePuy International, Leeds, UK) in every type of ankle arthritis (that is, post-traumatic, osteoarthritis, or rheumatoid arthritis) ^83^.

Newer-generation alignment guides for TAA have allowed greater accuracy and reproducibility in the placement of the implant components. The comparison of extramedullary and intramedullary referencing for tibial component alignment has shown greater accuracy for the latter for tibial component alignment in the sagittal plane but no significant difference between the two techniques in coronal plane alignment ^84^.

Veljkovic *et al*. ^85^ described a new measure able to describe the sagittal relationship between the talus and tibial shaft, called lateral talar station (LTS), showed by weight-bearing lateral ankle radiographs. LTS presents good reliability and may help to better define the sagittal position of the talus following TAA. Similar measurement of the sagittal position was described by Lee *et al*., who concluded that sagittal malalignment of the talus following TAA was less likely with the Mobility Total Ankle System than with the Hintegra total ankle system (Newdeal, Lyon, France; Integra, Plainsboro, NJ, USA) ^86^.

An excessive deformity of the tibiotalar joint in the coronal plane has been described as a contraindication for TAA. For this, the coronal plane alignment can be restored to neutral up to a maximum of 15° of varus or valgus using multiple procedures, such as deltoid ligament release, posterior soft-tissue releases, or lateral ligament reconstruction. On the basis of severity of malalignment in the coronal plane, no significant difference was detected in clinical or functional outcomes. Similar conclusions were reported in a level I study ^87^ that showed that ankles with pre-operative coronal plane of more than 10° varus deformity compared with ankles with less than 10° had satisfactory results following TAA.

TAA and supramalleolar osteotomy (SMOT) are valid options for the treatment of varus osteoarthritis of the ankle: TAA corrects more effectively the talar position in all planes than SMOT ^88^. Patients who had a hind-foot arthrodesis (isolated subtalar arthrodesis or triple arthrodesis) treated with TAA experienced significant improvements in pain and function, whereas patients without a hind-foot arthrodesis experience inferior outcomes ^89^.

## Conclusions

Many controversies still exist in the field of lower limb joint arthroplasty. Paradoxically, many novelties have not resulted in clinically relevant improvement. Despite being good marketing tools, these novelties may produce more problems than they solve. Accurate planning and perfect execution of “classic” options remain the mainstay for long-term success.

## Abbreviations

ALTR, adverse local tissue reaction; BMI, body mass index; Co-Cr, cobalt-chromium; DVT, deep vein thrombosis; ERAS, Enhanced Recovery After Surgery; KOOS, Knee injury Osteoarthritis Outcome Score; LTS, lateral talar station; PE, pulmonary embolism; PJI, periprosthetic joint infection; SMOT, supramalleolar osteotomy; TAA, total ankle arthroplasty; THA, total hip arthroplasty; Ti, titanium; TKA, total knee arthroplasty; TXA, tranexamic acid; VAS, visual analogue scale; VTP, venous thromboembolic prophylaxis
